# Pediatric Pneumonia Death Caused by Community-acquired Methicillin-Resistant *Staphylococcus aureus*, Japan

**DOI:** 10.3201/eid1408.070391

**Published:** 2008-08

**Authors:** Takashi Ito, Makiko Iijima, Takayoshi Fukushima, Masato Nonoyama, Masahiro Ishii, Tatiana Baranovich, Taketo Otsuka, Tomomi Takano, Tatsuo Yamamoto

**Affiliations:** * Kitasato University School of Medicine, Kanagawa, Japan; † Niigata University Graduate School of Medical and Dental Sciences, Niigata, Japan

**Keywords:** necrotizing pneumonia, Panton-Valentine leukocidin, community-acquired resistant Staphylococcus aureus (MRSA), letter

**To the Editor:** Community-acquired methicillin-resistant *Staphylococcus aureus* (CA-MRSA), which carries genes for Panton-Valentine leukocidin (PVL), has become a major concern worldwide (*1*–*3*). CA-MRSA is mainly associated with skin and soft tissue infections in young, otherwise healthy, persons in the community (*3*) and also with life-threatening sepsis and community-acquired pneumonia (preceded by influenza) (*1*,*3*,*4*). The role of PVL in the pathogenesis of staphylococcal infections is controversial. Whereas Labandeira-Rey et al. (*5*) provided data that PVL, in combination with staphylococcal protein A, destroys respiratory tissue and bacteria-engulfing immune cells, Voyich et al. (*6*) and Bubeck Wardenburg et al. (*7*) showed that PVL was not essential for the pathogenesis of skin disease, sepsis, or pneumonia in a mouse model.

Several types of CA-MRSA clones exist, e.g., CA-MRSA belonging to multilocus sequence type (ST) 1 (called the USA400 clone) and ST8 (called the USA300 clone), which have been major clones in North America (recently, USA300 is becoming more prominent); CA-MRSA belonging to ST80, which has been a major clone in Europe; and CA-MRSA belonging to ST30, which is distributed worldwide, including Japan (*2*,*8*). MRSA carrying the PVL gene (a marker of CA-MRSA [ST30]) comprises 0.1% of MRSA isolated in hospitals in Japan (*9*).We describe a fatal case of pediatric pneumonia and septic shock from CA-MRSA in Japan.

A 16-month-old, previously healthy boy was admitted to the hospital for fever and shortness of breath on August 30, 2006. He had had cold-like symptoms for 14 days and fever for the 2 previous days. On examination, hordeolum of the right eyelid and cyanosis were observed; the patient’s blood pressure was 106 (undetectable)/ mm Hg tachycardia 185 beats/min, tachypnea 72 breaths/min, and temperature 39.8°C. He had bilateral coarse breath sounds, and bronchovesicular breath sounds over the right lung. Chest radiography indicated lobar consolidation and pleural effusion on the right side. Laboratory analysis showed leukocytopenia, thrombocytopenia, elevated C-reactive protein level, and hypoxemia.

Intravenous administration of sulbactam/ampicillin and cefotaxime, and oxygen inhalation was started. Oxygen saturation did not improve, and laboratory values of disseminated intravascular coagulation (DIC) were observed: platelet count 121 K/mm^3^, fibrinogen level 528 mg/dL, fibrin degradation products 37.7 μg/mL, prothrombin time 1.86 international normalized ratio, and D-dimer 37.7 μg/mL. The condition was considered septic shock, and consequently the boy was transferred to the pediatric intensive care unit, where he required intubation and mechanical ventilation.

Sulbactam/ampicillin was switched to meropenem, and cefotaxime was continued. On day 2 after admission, chest radiography showed bilateral consolidation. On day 3, blood culture yielded MRSA, and cefotaxime was changed to vancomycin. Meropenem therapy was continued to cover possible mixed bacterial infection. Immunoglobulin therapy and DIC syndrome treatment (nafamostat mesilate, ulinastatin, freeze-dried concentrated human antithrombin III) were also started. On day 4, computed tomographic examination detected pneumothorax and athelectasis. Because laboratory data confirmed the presence of only MRSA, meropenem was changed to flomoxef (which belongs to the oxacephem family of β-lactam antimicrobial agents) on the expectation that a possible synergistic effect of flomoxef and vancomycin might occur. No major changes occurred on days 5 and 6. On day 7, in addition to bilateral infiltrates on chest radiography, the oxygen index was 65 (partial pressure of arterial oxygen/fraction of inspired oxygen), and the patient was considered to have acute respiratory distress syndrome. A percutaneous cardiopulmonary support system (a portable heart-lung machine that provides temporary circulatory support) was used, but in spite of treatment, there was no improvement, and the child died on day 10 after admission (September 8). An autopsy was not performed.

Molecular characterization of MRSA isolated from the blood was performed as described previously (*8*,*9*). Isolated MRSA (strain NN32) was positive for PVL, belonging to ST30:*spa*19:staphylococcal cassette chromosome mec (SCC*mec*)IVa, and was resistant to only β-lactam antimicrobial agents (Table).

**Table:** Characteristics of PVL-positive MRSA strains reported in Japan since 2000*

Type, gene, or resistance		PVL-positive MRSA					
		Present strain		Previous strains			
		NN32		NN1	NN12, NN31	EB 00449	DB 00319
Types	CC	30		30	30	30	30
	ST	30		30	30	30	765
	*spa*	19		19	19	19	43
	*agr*	3		3	3	3	3
	SCC*mec*	IVa		IVc	IVc	IVc	IVx*
	Coagulase	IV		IV	IV	IV	IV
Toxins							
Leukocidins	*lukS-PV, lukF-PV†*	+		+	+	+	+
	*lukE-lukD*, *lukM*	–		–	–	–	–
Hemolysins	*hla, hlg, hld*	+		+	+	+	+
	*hlb*	–		–	–	–	+
	*hlg–v*	–		–	–	–	–
Staphylococcal enterotoxins	*sea*	–		–	–	–	+
	*tst, seb, sec, sed, see, seh, sej, sek, sep*	–		–	–	–	–
	*egc‡*	+		+	+	+	+
	*seu*	+		+	+	+	+
Exfoliative toxins	*eta, etb, etd*	–		–	–	–	–
Others	*set*	+		+	+	+	+
	*edin*	–		–	–	–	–
Adhesins							
	*icaA, sdrD, sdrE*	–		–	–	–	–
	*icaD, cna*,§ *eno, fnbA, fnbB, ebpS,* *clfA, clfB, fib, sdrC, bbp¶*	+		+	+	+	+
Drug resistance and penicillinase plasmid	Aminoglycosides				GEN# KAN#*		GEN** KAN** STR**
	Macrolides						ERY**
	Lincosamides						CLI**
	Tetracycline				TET#		
	Penicillinase plasmid (kb)	+ (33)		+ (33)	+ (33)	+ (33)	+ (40)**

*PVL, Panton-Valentine leukocidin; MRSA, methicillin-resistant *Staphylococcus aureus*; SCC*mec* IVx, staphylococcal cassette chromosome *mec* type IV with unknown subtypes; +, positive; ­­–, negative; GEN, gentamicin; KAN, kanamycin; STR, streptomycin; ERY, erythromycin; CLI, clindamycin; TET, tetracycline. †GenBank accession nos. for the PVL gene sequences from strains NN32 and NN1 are AB286959 and AB186917, respectively. ‡*egc*, enterotoxin gene cluster, including *seg*, *sei*, *sem*, *sen*, and *seo* genes. §*cna*, gene encoding for collagen binding protein (adhesin). ¶*bbp*, gene encoding for bone sialoprotein binding protein (adhesin). #Drug resistance encoded by a conjugative drug resistance plasmid (pGKT1) (*8*). **Multidrug-resistant penicillinase plasmid encoding for resistance to GEN, KAN, STR, ERY, and CLI, in addition to penicillin resistance (*9*).

To date, all cases of PVL-positive CA-MRSA infections officially reported in Japan were caused by strains belonging to ST30 (*9*). All these strains can be classified into 2 types on the basis of *spa* type (Table), for example, ST30:*spa*19:SCC*mec*IVc. This type includes strain NN1, isolated from an 11-month-old patient with bullous impetigo (*8*); strain NN12, isolated from a 17-year-old patient with cutaneous abscess/osteomyelitis (*8*); strain NN31, isolated from an 18-year-old patient with pelvic abscesses (*9*); and strain EB00449, isolated from a 27-year-old patient with cutaneous abscesses (*9*). Another type is ST765 (single locus variant of ST30):*spa*43:SCC*mec*IVx. This type includes strain DB00319, isolated from a 61-year-old hospital inpatient (*9*).

The molecular characteristics of strain NN32 were similar to those of strain NN1, except for SCC*mec*IV subtypes (Table). Moreover, pulsed-field gel electrophoresis patterns (data not shown) and the PVL gene sequences of the 2 strains (NN32 and NN1) were identical (Table).

This case of CA-MRSA ST30 infection in a child represents a progression from common cold–like symptoms (occurring outside the influenza season) to fatal pneumonia, despite intensive therapy, including the administration of sensitive antimicrobial agents. CA-MRSA ST30 contains several genes that mediate adhesion (e.g., *cna* and *bbp*) and toxin genes (PVL and *egc*, which encode for at least 5 superantigens, including staphylococcal enterotoxin G, I, M, N, and O). The gene cluster *egc* is associated with septic shock (*10*). Further studies are needed to clarify the pathogenesis of community-acquired pneumonia caused by CA-MRSA.
